# Supplementary material for: BRCA2 deficiency instigates cGAS-mediated inflammatory signaling and confers sensitivity to tumor necrosis factor-alpha-mediated cytotoxicity
Source: Nat Commun. 2019 Jan 9;10:100. doi: 10.1038/s41467-018-07927-y (PMC6327059; doi:10.1038/s41467-018-07927-y)

Supplementary Information file

BRCA2 deficiency instigates cGAS-mediated inflammatory signaling and confers sensitivity to Tumor Necrosis Factor-alpha-mediated cytotoxicity.

Heijink et al.

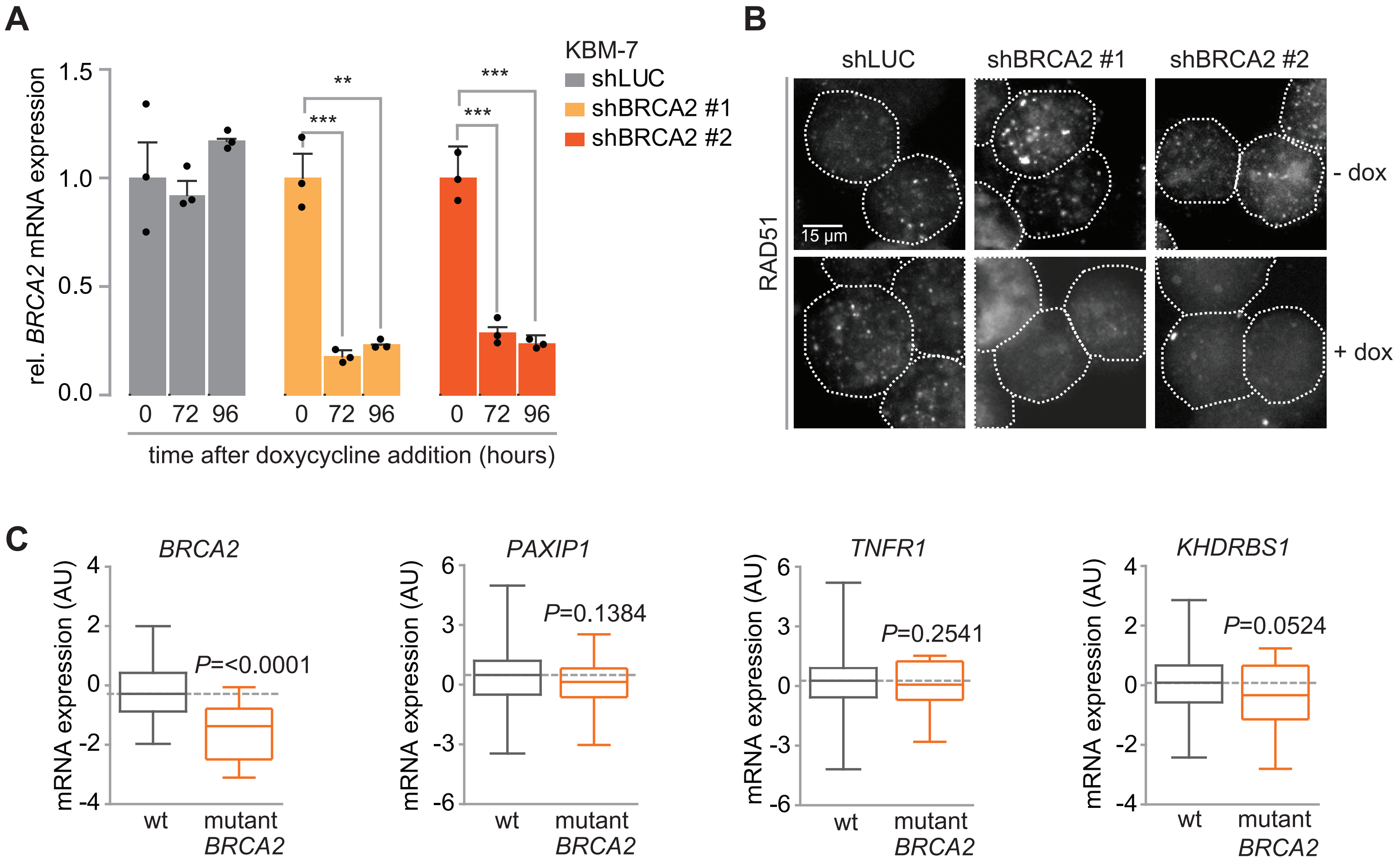

Supplementary Figure 1. BRCA2 deficiency of KBM-7-shBRCA2 cell lines.
(a) KBM-7 cells harboring indicated shRNAs were treated with doxycycline for indicated time periods. BRCA2 mRNA expression was measured using RT-qPCR. Measurements were normalized to expression levels at day 0. Error bars represent s.d. of three independent measurements. P values were calculated using two-tailed Student's t-test. ** indicates P<0.01, *** indicates P<0.001. **(b)** Representative immunofluorescence images of KBM-7 cells harboring indicated hairpins and treated with or without doxycycline for four days. Cells were irradiated (5 Gy) and stained three hours post irradiation with anti-RAD51 and DAPI. **(c)** mRNA expression levels of indicated genes were assessed in BRCA2 wt (n=193) and germline BRCA2 mutant (n=23) serous ovarian cancer cases from the TCGA dataset.

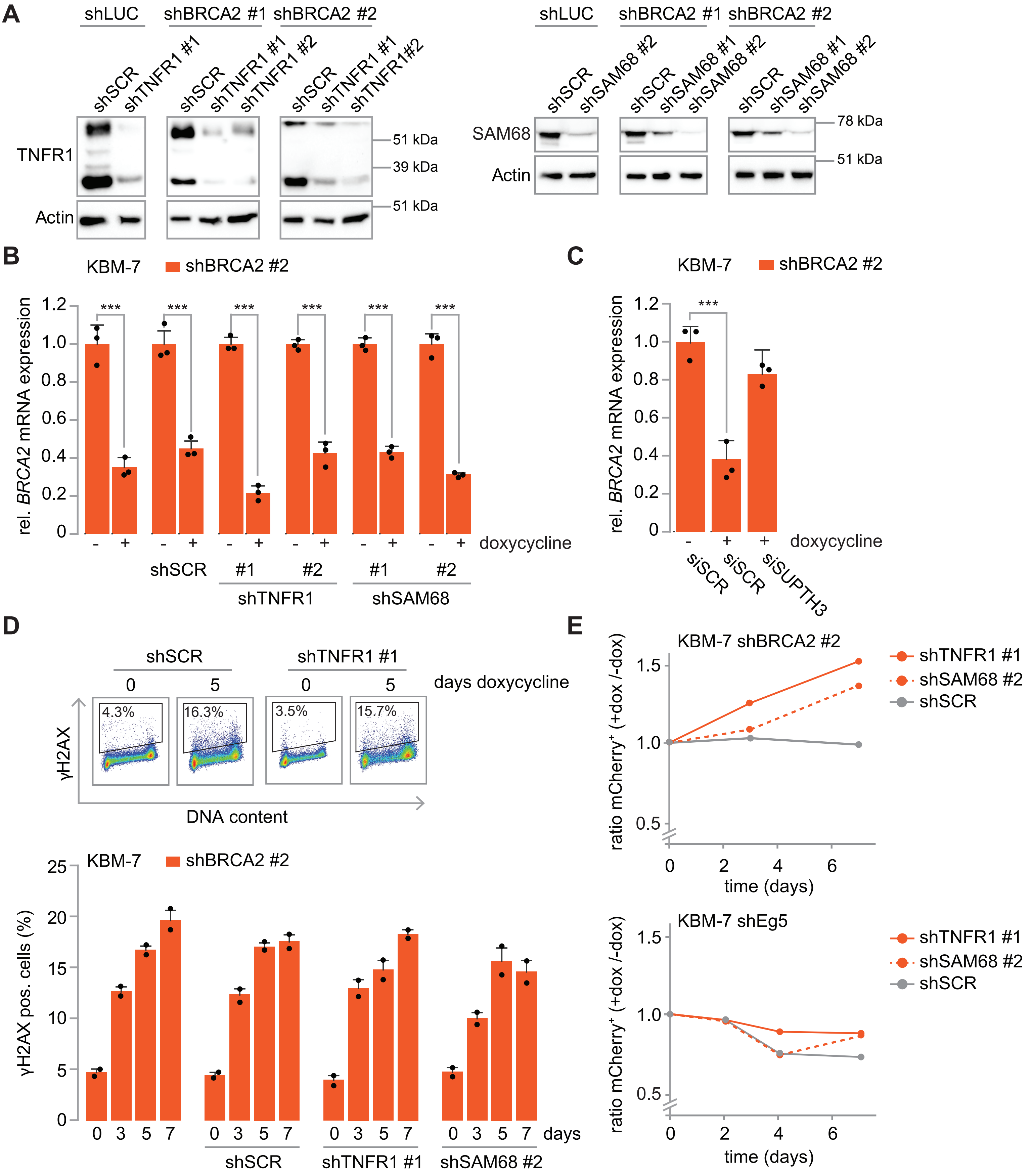

Supplementary Figure 2. Loss of TNFR1 or SAM68 specifically rescues BRCA2 deficiency.
(a) pLKO.tet.shRNA transduced KBM-7 cells, which were additionally infected with indicated shRNA vectors carrying IRES-driven mCherry cassettes. Cells were treated with doxycycline for four days prior to cell lysis, and immunoblotted for TNFR1, SAM68 or Actin. **(b)** KBM-7 cells transduced with indicated hairpins were treated with or without doxycycline for four days. BRCA2 mRNA expression was measured using RT-qPCR. Measurements were normalized to expression levels without doxycycline treatment. Error bars represent s.d. of three independent measurements. P values were calculated using two-tailed Student's t-test. *** indicates P<0.001. **(c)** KBM-7-shBRCA2 #2 cells were transfected with indicated siRNAs and simultaneously treated with doxycycline for three days. BRCA2 mRNA expression was measured and quantified as for panel (b). **(d)** γH2AX levels were measured by flow cytometry. Indicated KBM-7 cell lines were treated with doxycycline for indicated time periods prior to fixation. Gating was performed as shown in the upper panels. **(e)** KBM-7-pLKO.tet.shBRCA2 #2 or KBM-7-pLKO.tet.shEg5 cells carrying mCherry shRNA cassettes for TNFR1, SAM68 or SCR were treated with or without doxycycline. Percentages of mCherry-positive cells were measured every two or three days after start of doxycycline treatment. Ratios of mCherry-positive cells in doxycycline treated cultures versus untreated cultures are indicated. Per condition, at least 30,000 events were measured.

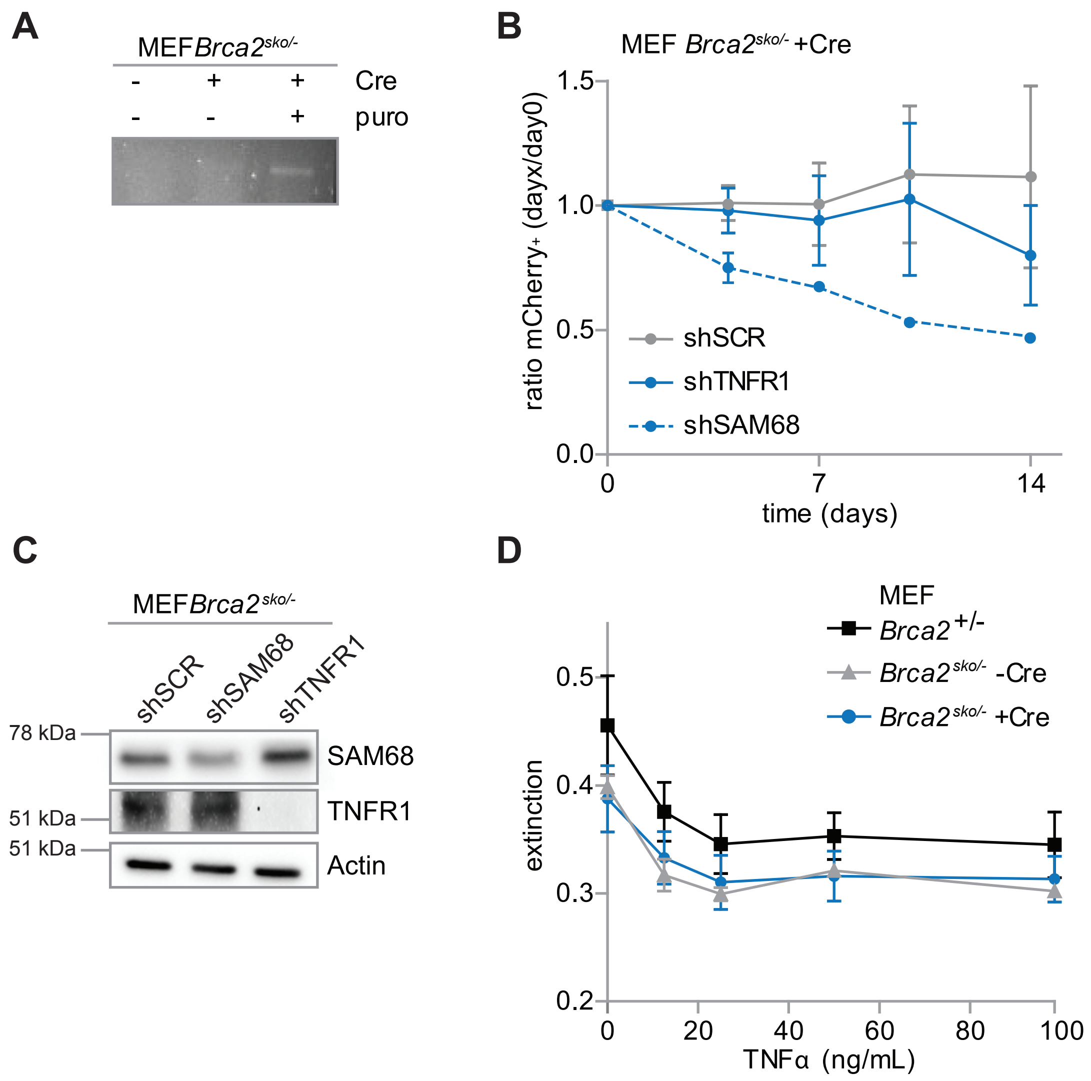

Supplementary Figure 3. TNFα signaling does not interfere with viability of Brca2-depleted MEFs.
(a) Brca2$^{sko/-}$ MEFs were infected with Cre retrovirus and subsequently selected with puromycin for two days (2 µg per mL). Genomic DNA was isolated and Cre targeting of the Brca2^{sko} allele was detected by genomic PCR (product: 110 base pairs). **(b)** Brca2$^{sko/-}$ MEFs were infected with mCherry-expressing hairpins against TNFR1, SAM68 or scrambled sequence (SCR). Cells were treated with Cre recombinase and percentages of mCherry-positive cells were measured using flow cytometry. The ratios of mCherry-positive cells at day x compared to percentages of mCherry-positive at day 0 cells were calculated. Error bars represent s.d. of three independent experiments. **(c)** Immunoblotting of Brca2$^{sko/-}$ MEFs infected with mCherry-expressing hairpins against SAM68 or TNFR1. **(d)** Indicated MEF lines were plated and treated with TNFα for five days. Cell viability was assessed by MTT conversion. Error bars represent s.d. of three technical replicates.

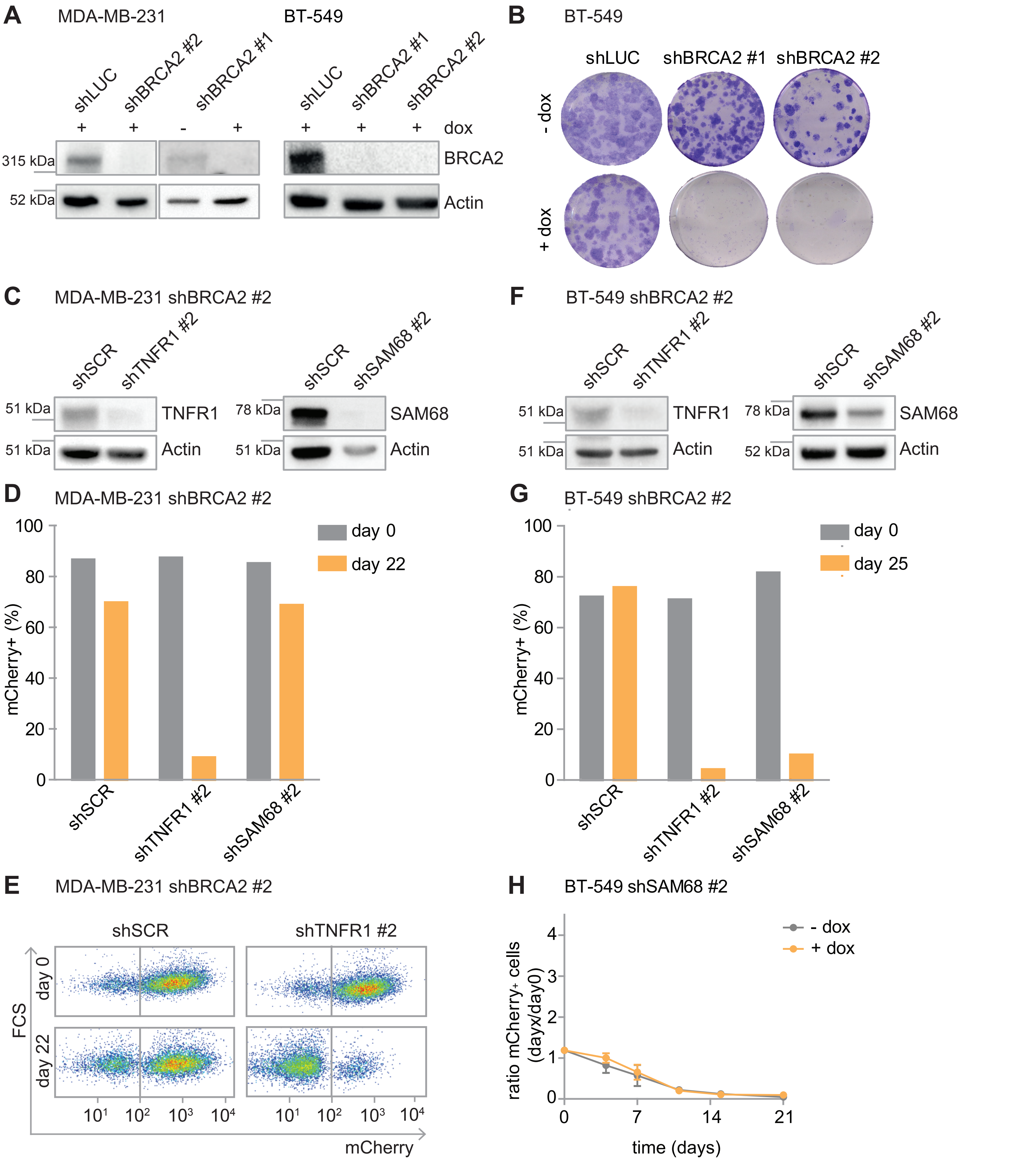

Supplementary Figure 4. Loss of TNFR1 and SAM68 in BT-549 and MDA-MB-231 affects cell viability.
(a) MDA-MB-231 and BT-549 cells harboring shLUC, shBRCA2 #1 or shBRCA2 #2 were treated with or without doxycycline for four days and immunoblotted for BRCA2 and Actin. **(b)** BT-549 cells harboring indicated hairpins were plated in 6-well plates and treated with or without doxycycline. Cells were fixed after 14 days and stained with crystal violet. **(c)** Immunoblotting of MDA-MB-231-shBRCA2 #2 cells infected with shTNFR1 #2, shSAM68 #2 or shSCR. **(d)** Quantification of mCherry-positive MDA-MB-231-shBRCA2 #2 cells, after transduction with mCherry-expressing shTNFR1 #2, shSAM68 #2 or shSCR after 22 days. **(e)** Representative flow cytometry plots of MDA-MB-231-shBRCA2 #2 with mCherry-expressing shTNFR1 #2 or shSCR, treated for 22 days without doxycycline. Cells were gated on mCherry positivity. **(f)** Immunoblotting of BT-549-shBRCA2 #2 cells infected with shTNFR1 #2, shSAM68 #2 or shSCR.
(g) Quantification of mCherry-positive BT-549-shBRCA2 #2 cells, after transduction with mCherry-expressing shTNFR1 #2, shSAM68 #2 or shSCR after 25 days. **(h)** BT-549-shBRCA2 #2 cells harboring mCherry-expressing shSAM68 #2 were treated with or without doxycycline for indicated time periods. mCherry positivity was measured by flow cytometry. Percentages were normalized to mCherry percentages at day 0.

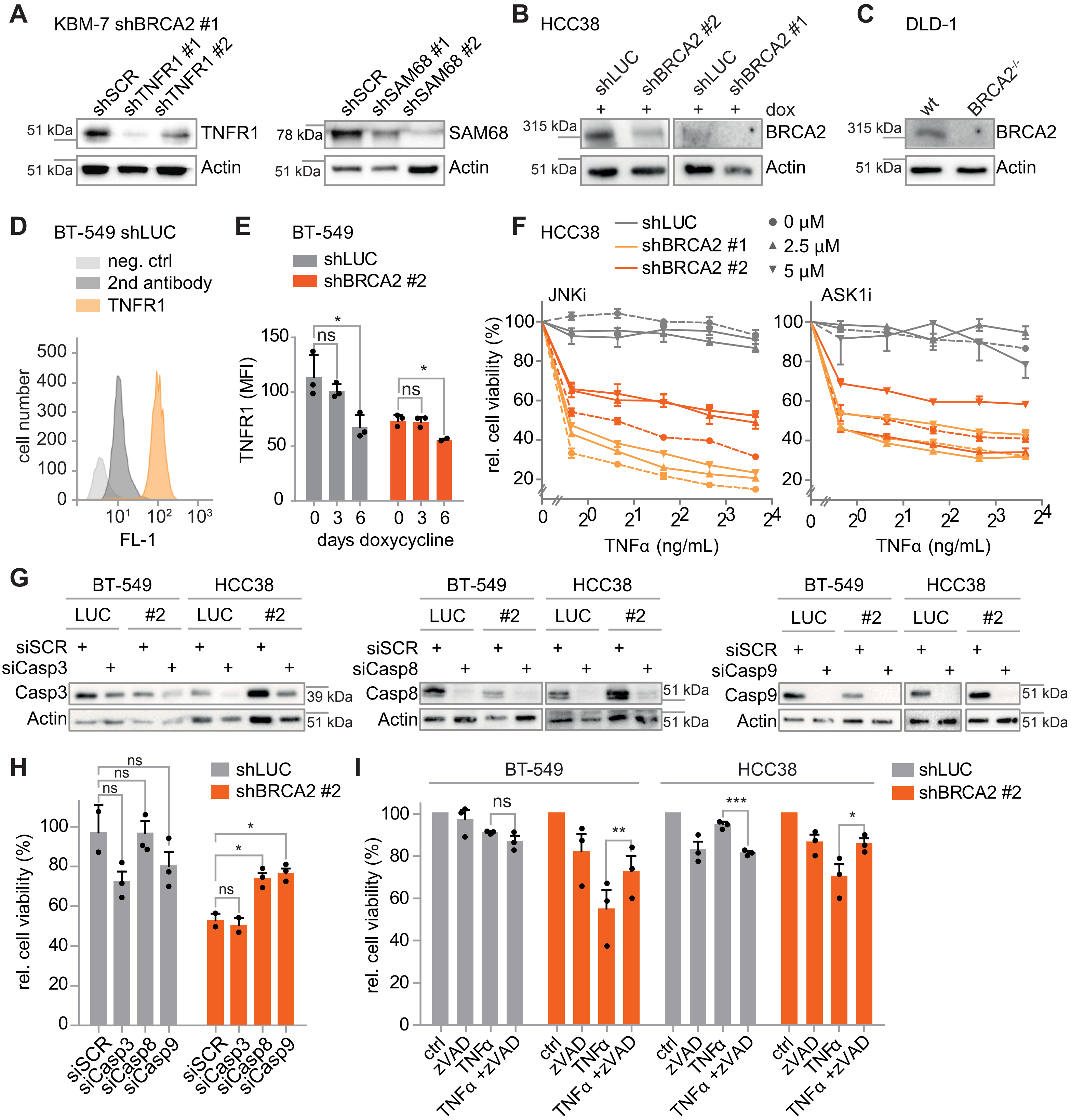

Supplementary Figure 5. TNFα sensitivity is ASK1/JNK and caspase dependent and is not due to changes of TNFR1 expression levels. (a) Immunoblotting of KBM-7 shBRCA2 #1 cells with indicated hairpins against SAM68 and TNFR1. **(b)** Immunoblotting of HCC38 cells harboring shLUC or shBRCA2 #2 treated with doxycycline for four days. **(c)** Immunoblotting of DLD-1 wt and BRCA2-/- cells for BRCA2 and Actin. **(d)** Representative flow cytometry histogram of BT-549-shLUC cells stained with anti-TNFR1. **(e)** BT-549 cells harboring shLUC or shBRCA2 #2 were treated with doxycycline for the indicated time periods. Expression of TNFR1 was measured using flow cytometry as in panel (d). Mean MFI of three independent experiments is indicated. P values were calculated using two-tailed Student's t-test. * indicates P<0.05. **(f)** HCC38 cells harboring shLUC, shBRCA2 #1 or shBRCA2 #2 were treated with doxycycline for 48 hours and subsequently treated with indicated concentrations of TNFα and JNK inhibitor (left panel) or ASK1 inhibitor (right panel) for five days. **(g)** Knockdown efficiencies of siRNAs targeting caspase-3, caspase-8 or caspase-9. BT-549 and HCC38 cells carrying shLUC (LUC) or shBRCA2 #2 (#2) were transfected with indicated siRNAs and harvested at 5 days after transfection. **(h)** HCC38 shLUC and shBRCA2 #2 cells were transfected with indicated siRNAs for 24-48 hours, and were subsequently treated with doxycycline for 48 hours. Cells were plated and treated with indicated TNFα concentrations for five days. **(i)** BT-549 and HCC38 shLUC or shBRCA2 #2 cells were treated with doxycycline for 48 hours and subsequently treated with zVAD-FMK (25 µM) and/or TNFα (12.5 ng per mL) for five days. For panels f, h and i, cell viability was assessed by MTT conversion. Error bars indicate s.e.m. of at least three independent experiments, with four technical replicates each. Measurements were normalized to untreated cells. P values were calculated using two-tailed Student's t-test. * indicates P<0.05, ** indicates P<0.01, *** indicates P<0.001. For P values, see Supplementary Data 4.

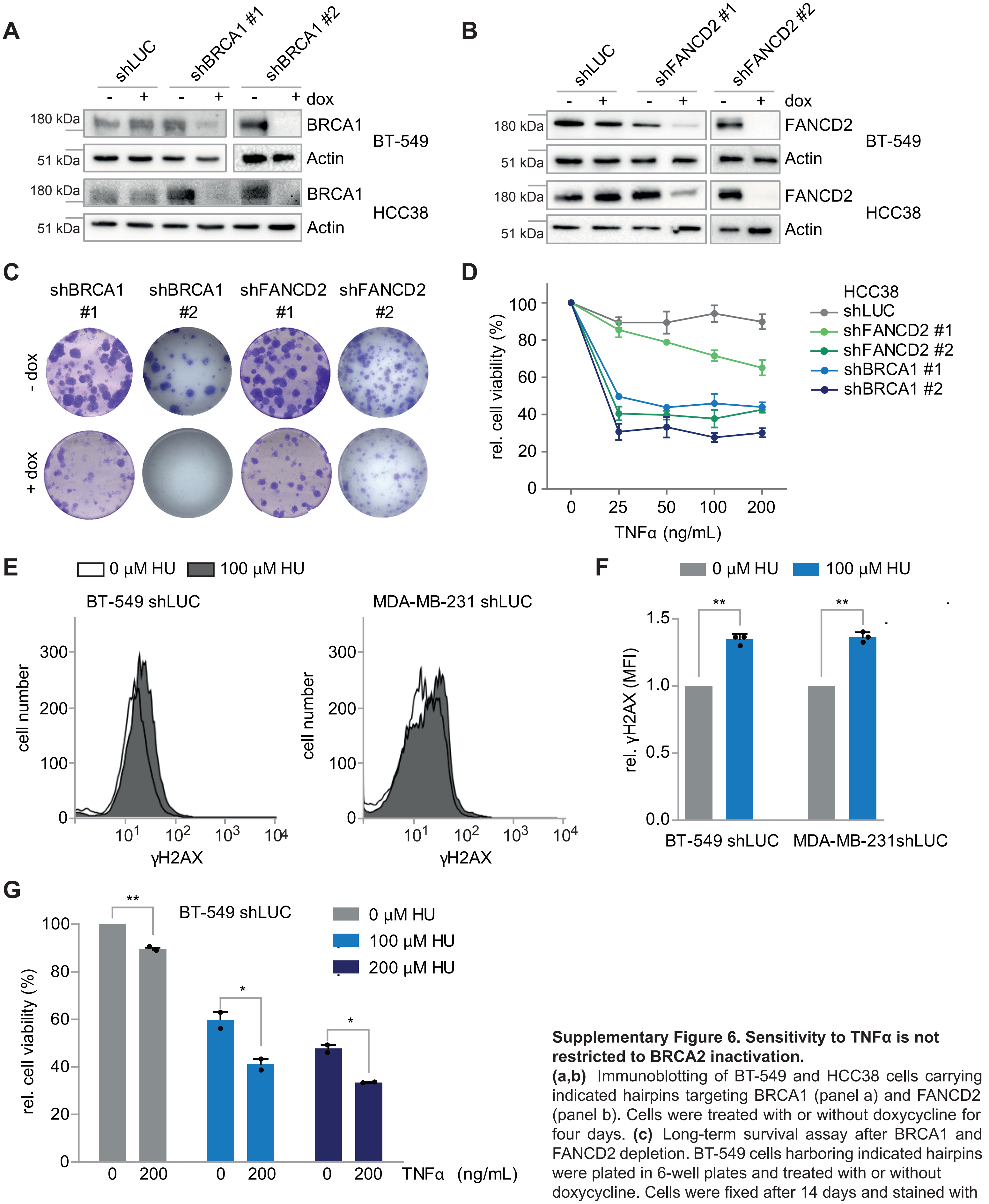

Supplementary Figure 6. Sensitivity to TNFα is not restricted to BRCA2 inactivation.
(a,b) Immunoblotting of BT-549 and HCC38 cells carrying indicated hairpins targeting BRCA1 (panel a) and FANCD2 (panel b). Cells were treated with or without doxycycline for four days. **(c)** Long-term survival assay after BRCA1 and FANCD2 depletion. BT-549 cells harboring indicated hairpins were plated in 6-well plates and treated with or without doxycycline. Cells were fixed after 14 days and stained with crystal violet. **(d)** HCC38 cells harboring hairpins against BRCA1 and FANCD2 were treated with doxycycline for 48 hours and subsequently plated and treated with indicated TNFα concentrations for five days. Cell viability was assessed by MTT conversion. Error bars indicate s.e.m. of three independent experiments, with three technical replicates each. For P values, see Supplementary Data 4. **(e)** Representative flow cytometry histograms of BT-549-shLUC and MDA-MB-231-shLUC cells treated with or without hydroxyurea (100 µM) for 24 hours and stained with anti-γH2AX. **(f)** Quantification of mean fluorescence intensity (MFI) of γH2AX staining in BT-549-shLUC and MDA-MB-231-shLUC cells as shown in panel(e). γH2AX MFI of cells treated with hydroxyurea was normalized to untreated cells. Error bars show s.d. of three independent experiments. P values were calculated using two-tailed Student's t-test. * indicates P<0.05, ** indicates P<0.01, *** indicates P<0.001. **(g)** BT-549-shLUC cells were plated and treated with or without indicated concentrations of hydroxyurea and TNFα for five days. Cell viability was assessed by MTT conversion. Error bars represent s.d. of two independent experiments. P values were calculated using two-tailed Student's t-test. * indicates P<0.05, ** indicates P<0.01, *** indicates P<0.001.

A BT-549

geneset	NES	FDR q-val
HALLMARK_KRAS_SIGNALING_UP	1.832848	0
HALLMARK_INTERFERON_ALPHA_RESPONSE	1.675349	0.004993
HALLMARK_INTERFERON_GAMMA_RESPONSE	1.622413	0.009621
HALLMARK_IL6_JAK_STAT3_SIGNALING	1.527058	0.027701
HALLMARK_COMPLEMENT	1.443326	0.056798
HALLMARK_KRAS_SIGNALING_DN	1.420548	0.062633
HALLMARK_INFLAMMATORY_RESPONSE	1.412346	0.058934
HALLMARK_TNFA_SIGNALING_VIA_NFKB	1.277374	0.222246
HALLMARK_COAGULATION	1.266338	0.216452
HALLMARK_ESTROGEN_RESPONSE_LATE	1.226254	0.285812

B HCC38

geneset	NES	FDR q-val
HALLMARK_TNFA_SIGNALING_VIA_NFKB	1.852496	0
HALLMARK_PROTEIN_SECRETION	1.766128	0
HALLMARK_KRAS_SIGNALING_UP	1.713606	3.07E-04
HALLMARK_TGF_BETA_SIGNALING	1.667727	4.75E-04
HALLMARK_COAGULATION	1.610957	0.001906
HALLMARK_APOPTOSIS	1.542571	0.005918
HALLMARK_ANGIOGENESIS	1.528939	0.007815
HALLMARK_INFLAMMATORY_RESPONSE	1.528897	0.006838
HALLMARK_COMPLEMENT	1.507485	0.00915
HALLMARK_INTERFERON_ALPHA_RESPONSE	1.494544	0.009999

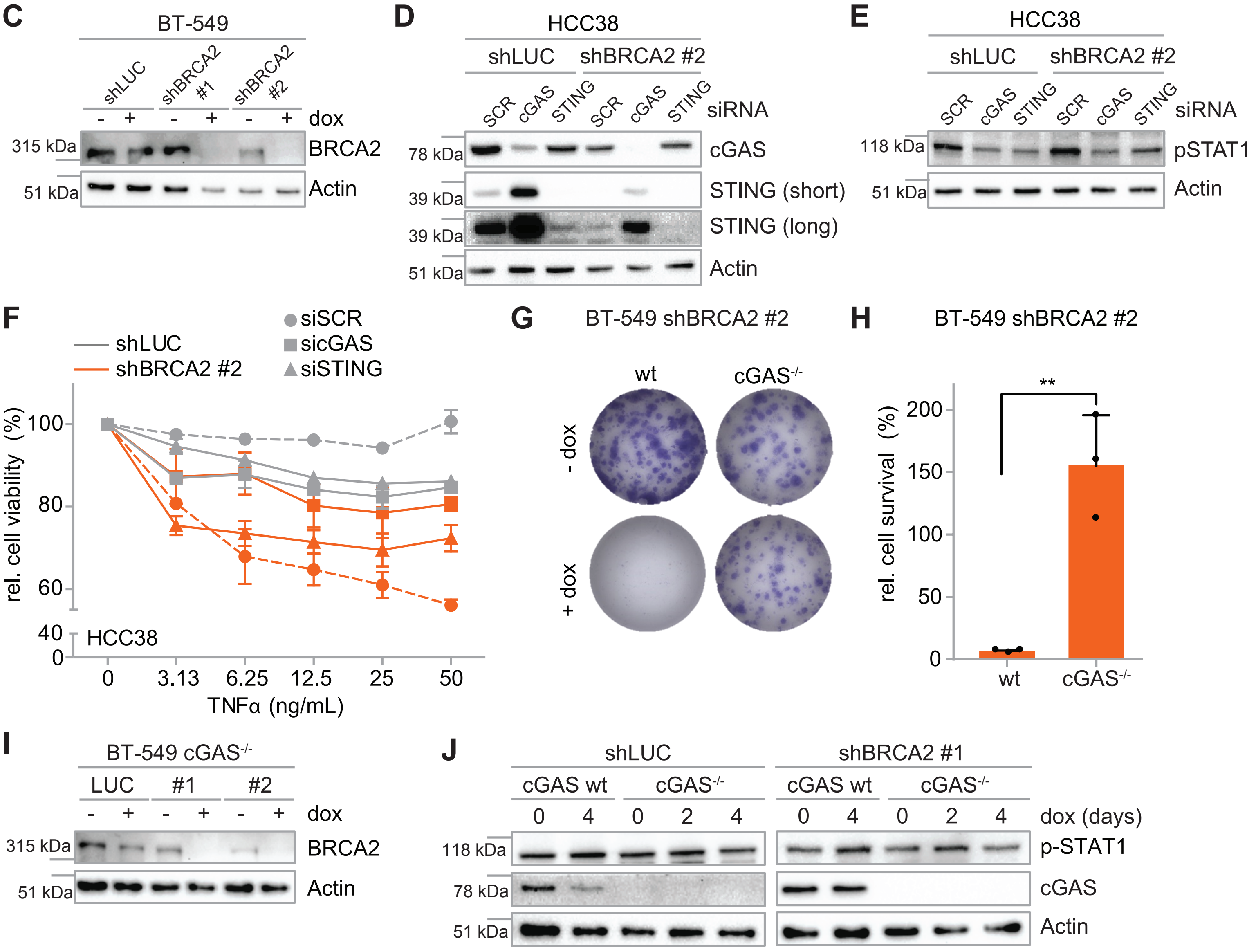

Supplementary Figure 7. BRCA2 depletion induces cGAS-STING dependent interferon signaling.
(a,b) Top 10 enriched Hallmark gene sets in BRCA2-depleted BT-549 (A) and HCC38 (B) cells compared to control cell lines. **(c)** BT-549 cells with indicated hairpins were treated with or without doxycycline for 6 days. Depletion of BRCA2 was confirmed by immunoblotting. **(d)** HCC38 cells with shLUC or shBRCA2 #2 were depleted for cGAS or STING. Knockdown efficiency of cGAS and STING was analyzed by immunoblotting five days post transfection. **(e)** HCC38 cells with indicated hairpins were depleted for cGAS or STING for 24 hours and subsequently treated with doxycycline for four days. Activation of STAT1 signaling was analyzed by immunoblotting. **(f)** HCC38 cells with indicated hairpins were depleted for cGAS or STING for 24 hours. Cells were plated and pre-treated with doxycycline for 48 hours and subsequently treated with indicated TNFα concentrations for five days. Cell viability was assessed by MTT conversion. Error bars indicate s.e.m. of three independent experiments with three technical replicates each. For P values, see Supplementary Data 4. **(g)** BT-549 cGAS$^{-/-}$ or wt cells harboring shBRCA2 #2 were plated in 6-well plates and treated with or without doxycycline. Cells were fixed after 11 days and stained with crystal violet. **(h)** Clonogenic cell survival as described in G was quantified. Percentage of cell survival was calculated by normalizing counted colonies to cells without doxycycline treatment. Error bars indicate s.d. of three independent experiments. P values were calculated using two-tailed Student's t-test. ** indicates P<0.01 **(i)** BT-549 cGAS$^{-/-}$ cells with indicated BRCA2 shRNAs or shLUC were treated with or without doxycycline for 4 days. Depletion of BRCA2 was confirmed by immunoblotting. **(j)** cGAS$^{-/-}$ or wt BT-549 cells with indicated hairpins were treated with doxycycline for indicated time periods. Expression of cGAS and the phosphorylation status of STAT1 were analyzed by immunoblotting.

Supplementary Figure 8 - Uncropped western blots

Figure 1A

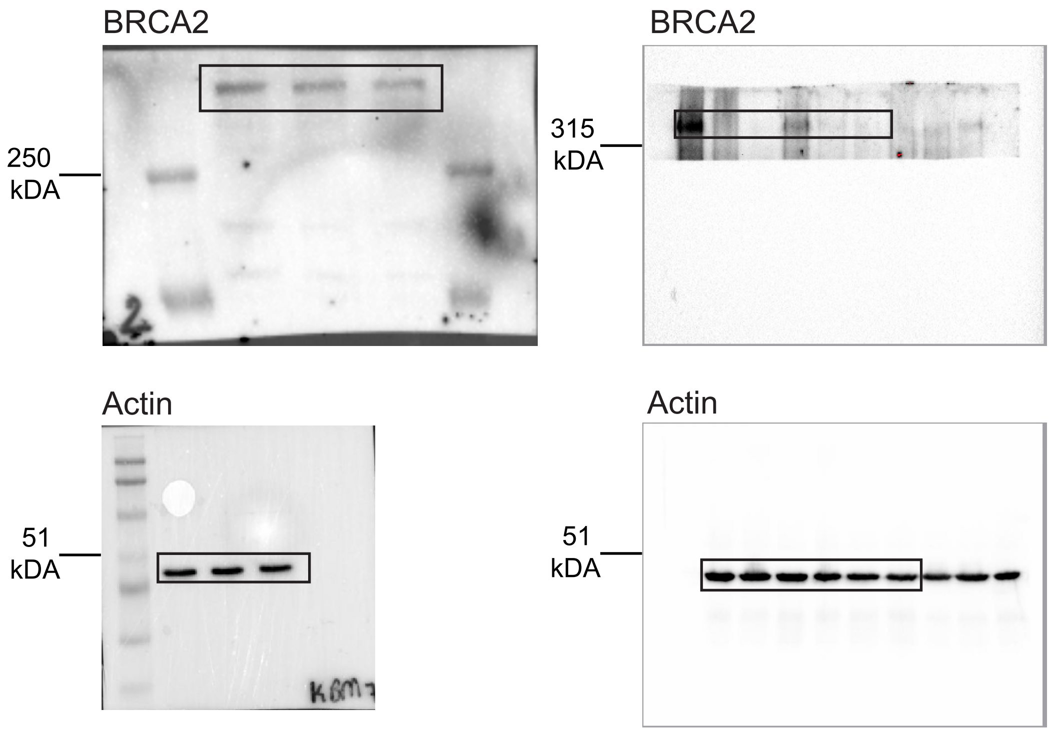

Figure 3C

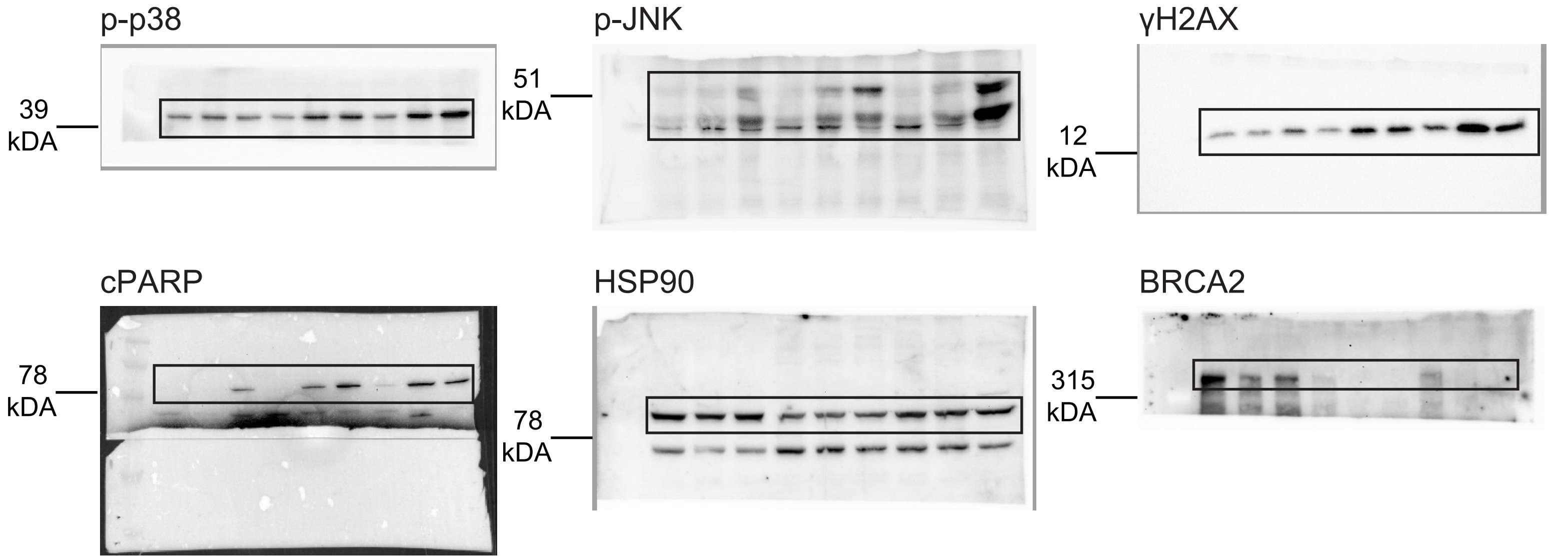

Figure 6D

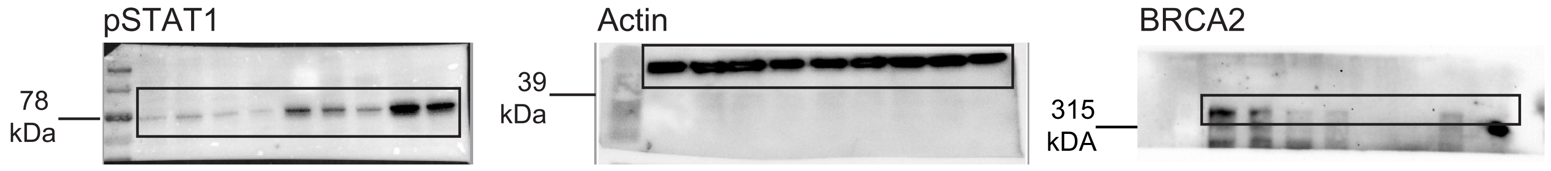

Supplementary Figure 9 - Uncropped western blots

Figure 6E

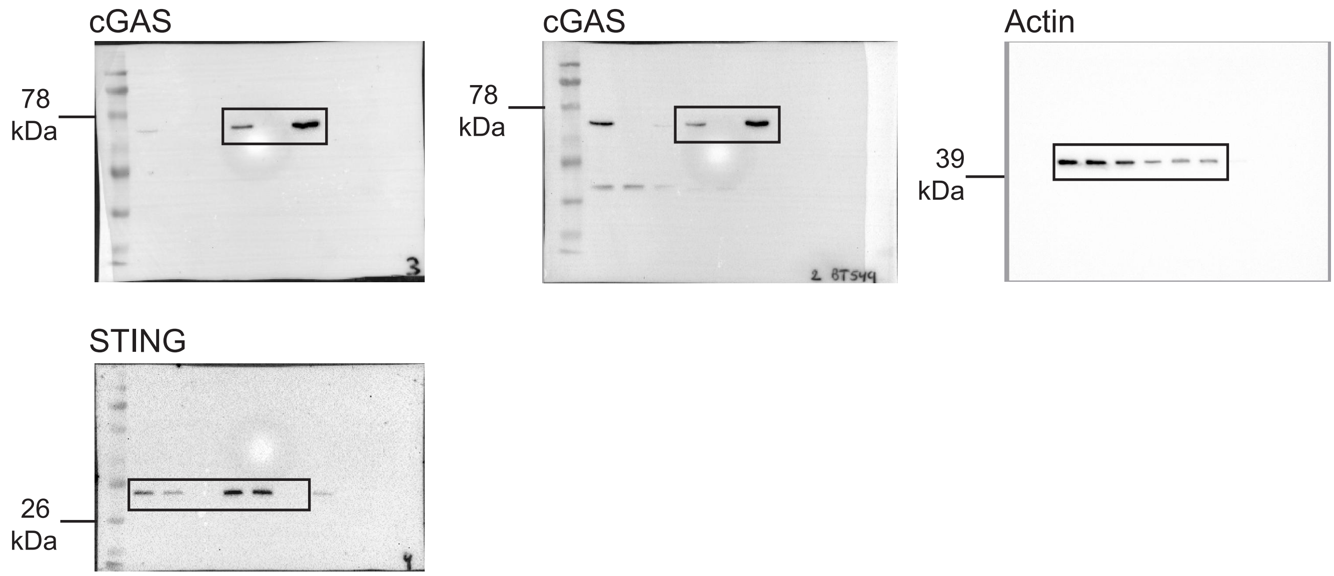

Figure 6F

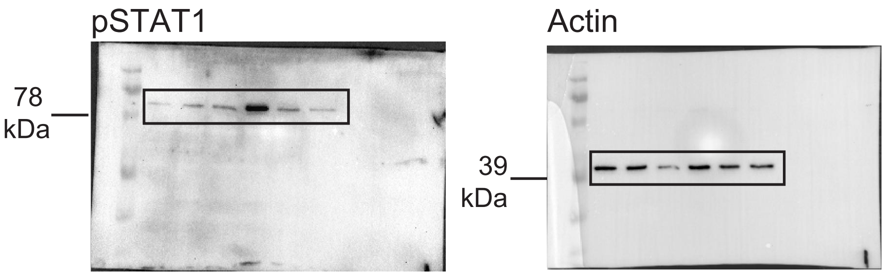

Figure 6G

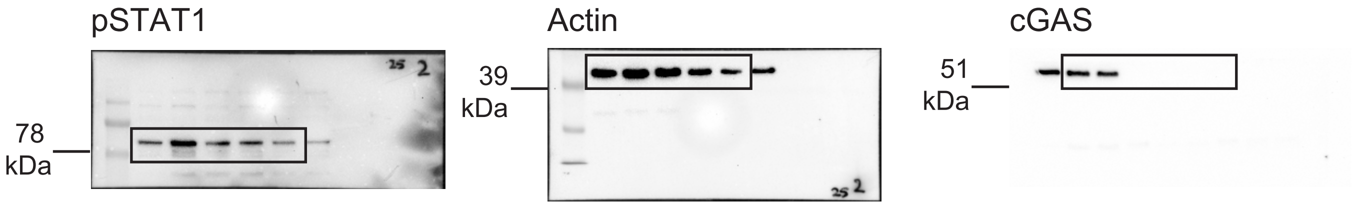

Figure S1D

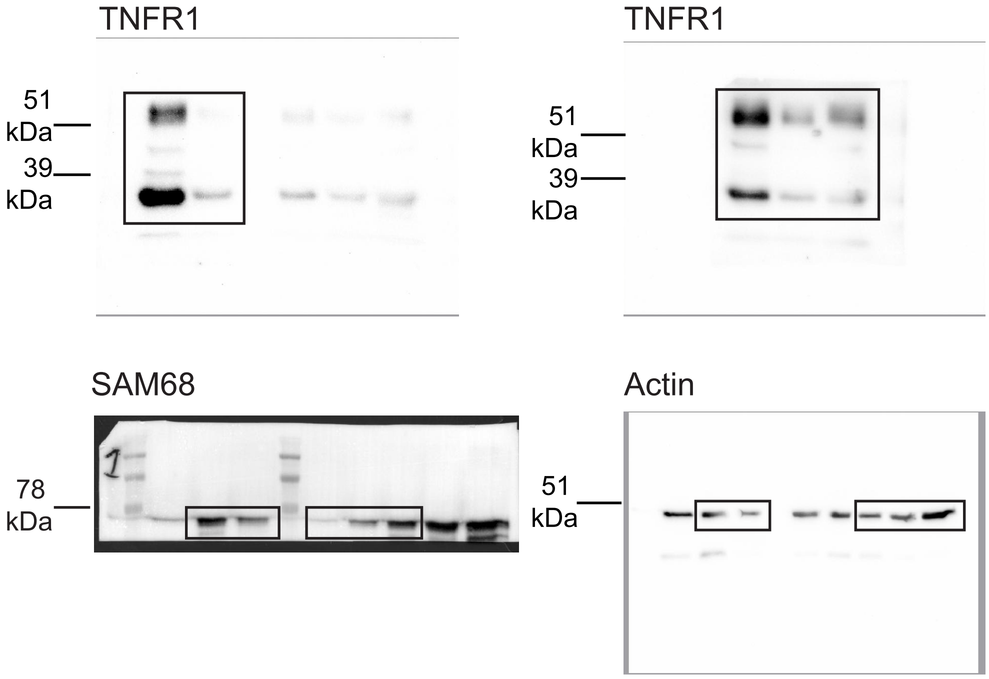

Supplementary Figure 10 - Uncropped western blots

Figure S3C

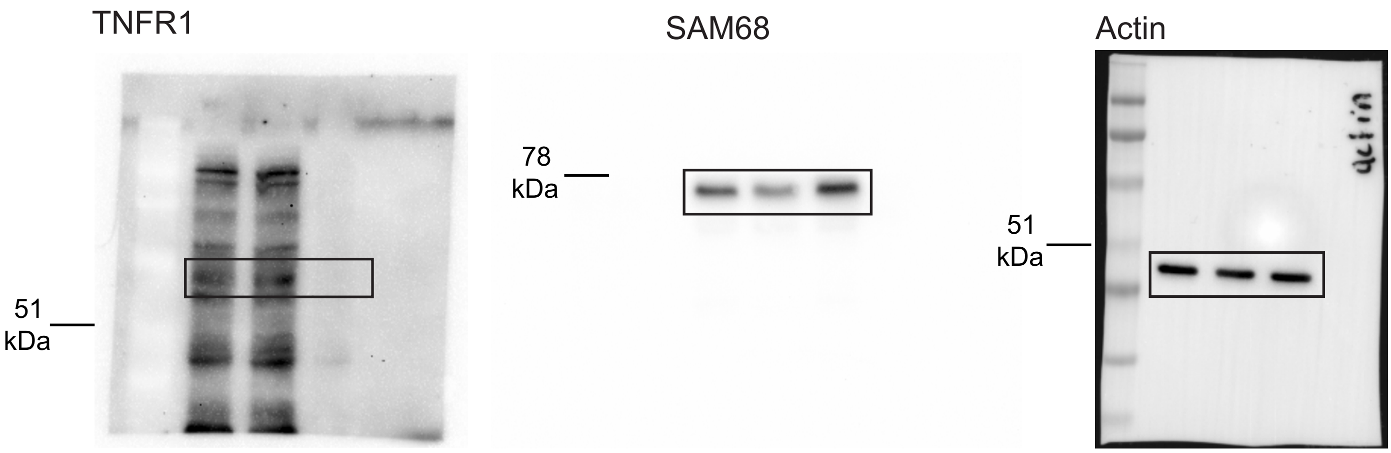

Figure S4A

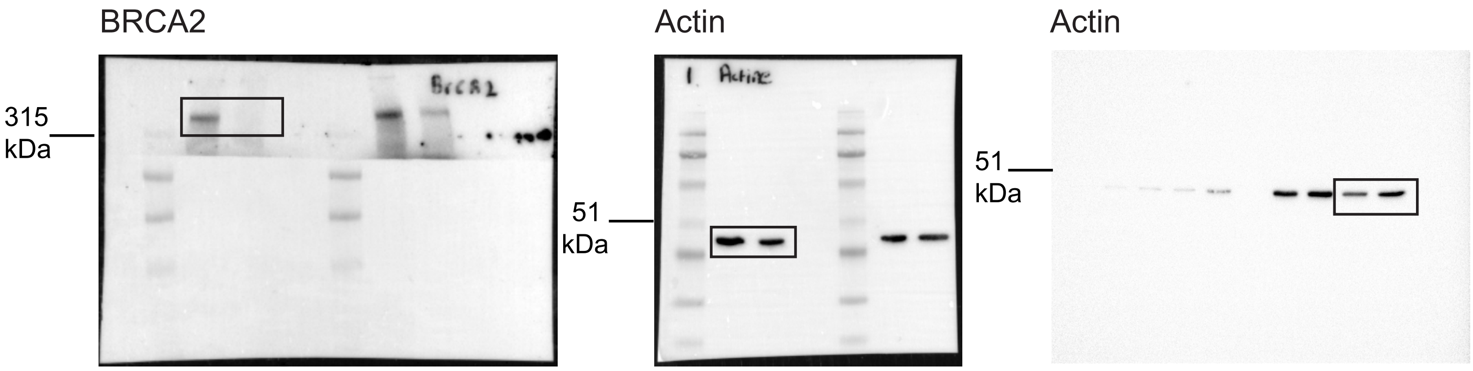

Figure S4C

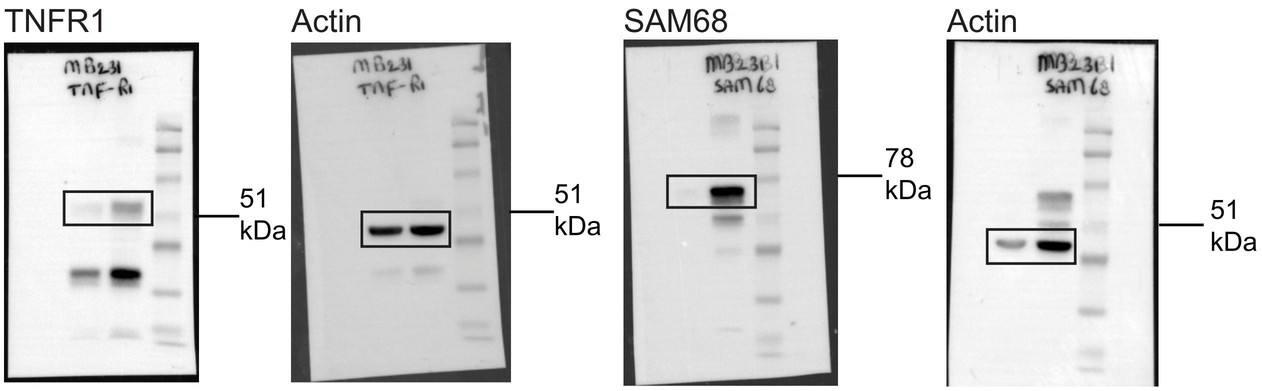

Figure S4F

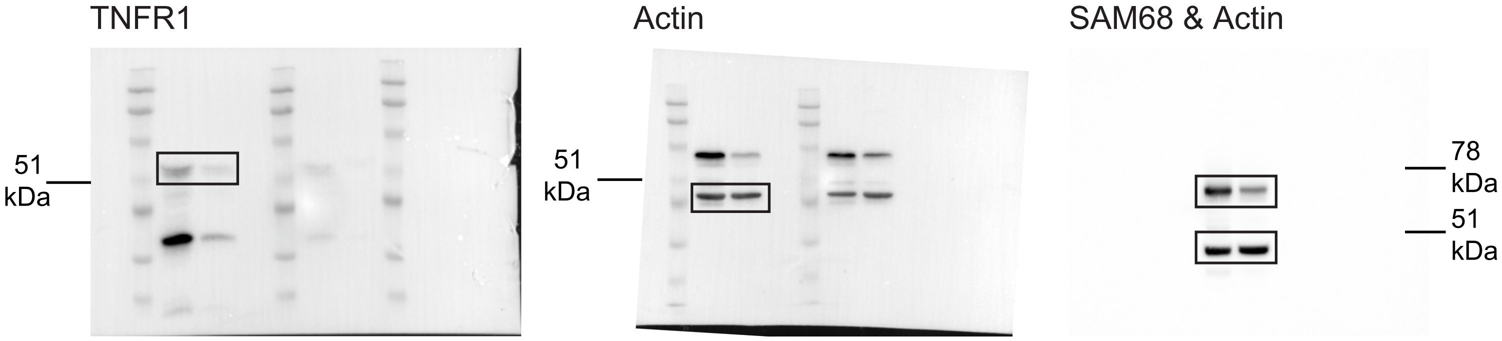

Supplementary Figure 11 - Uncropped western blots

Figure S5A

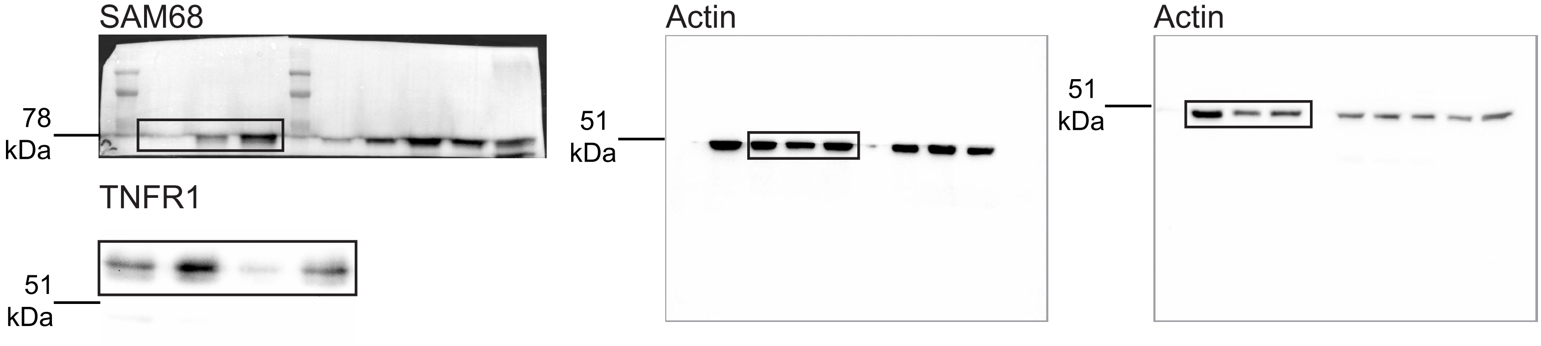

Figure S5B

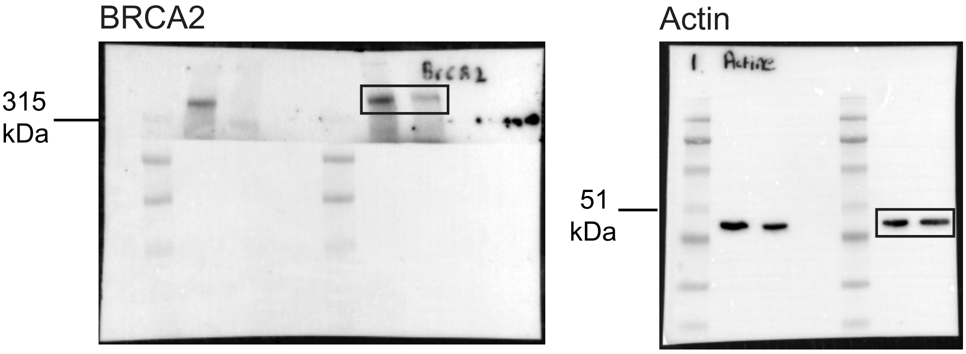

Figure S5C

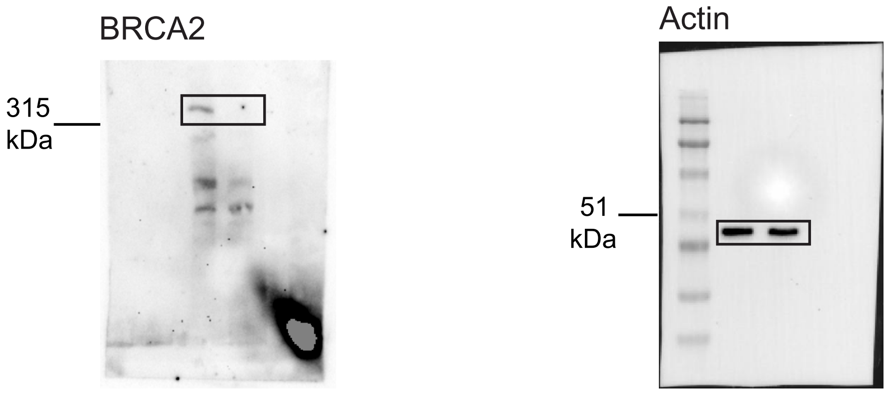

Figure S5H

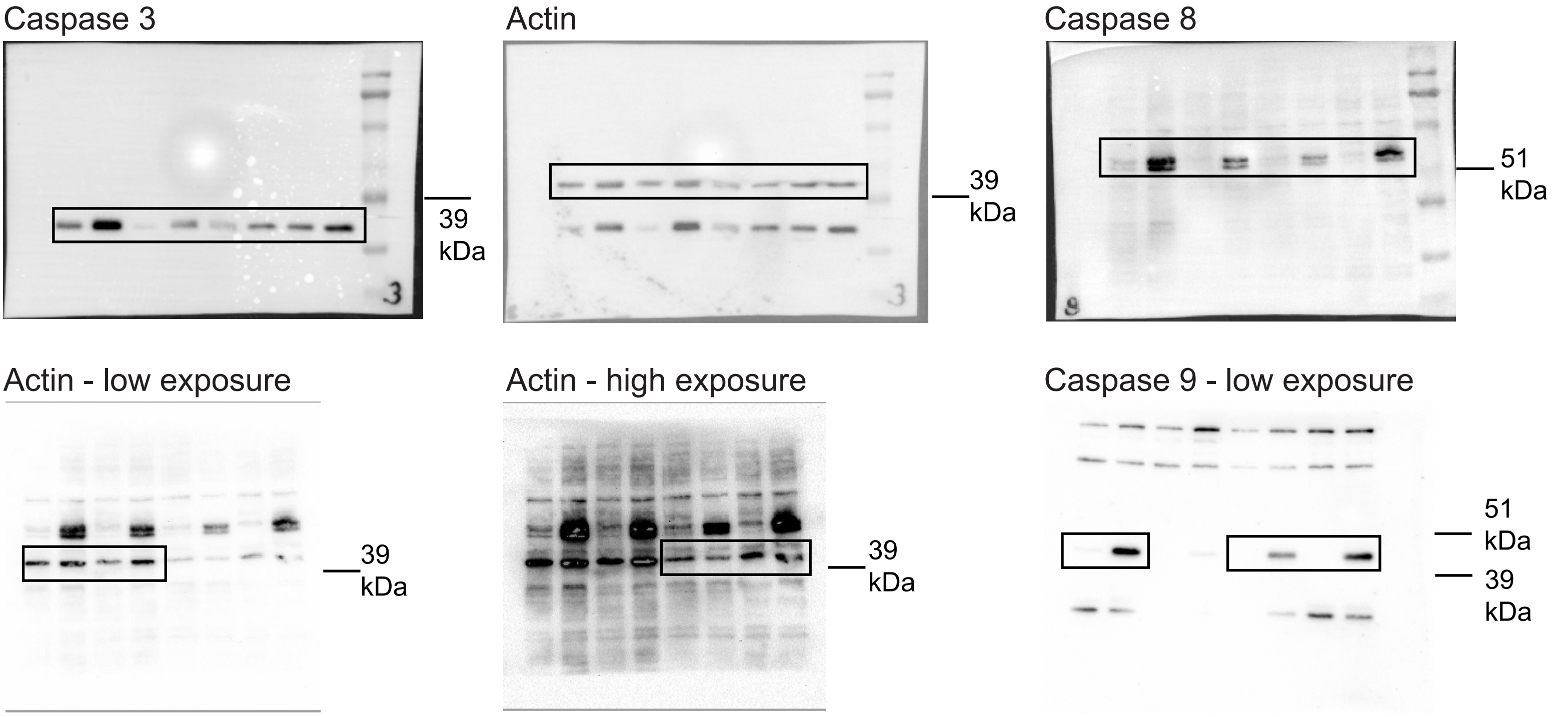

Supplementary Figure 12 - Uncropped western blots

Figure S5H

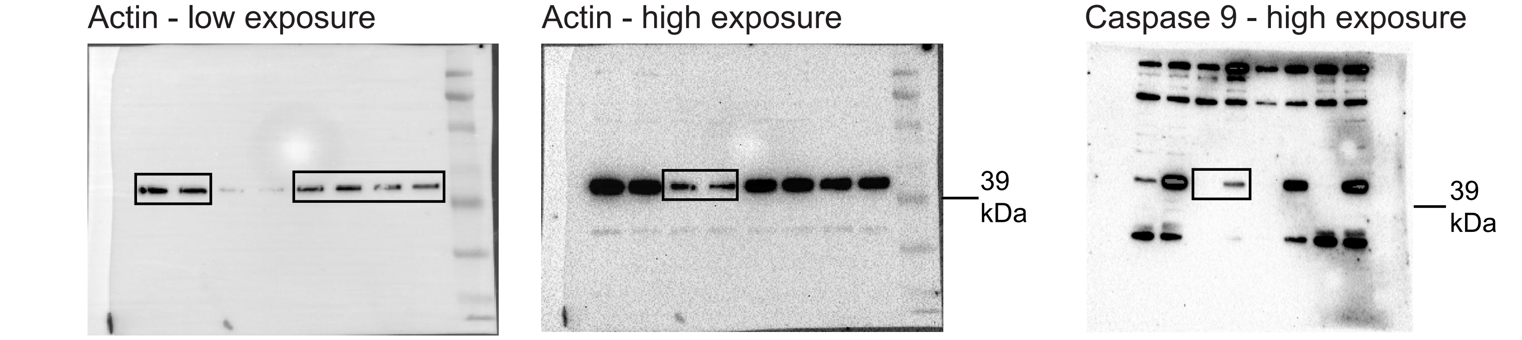

Figure S6A

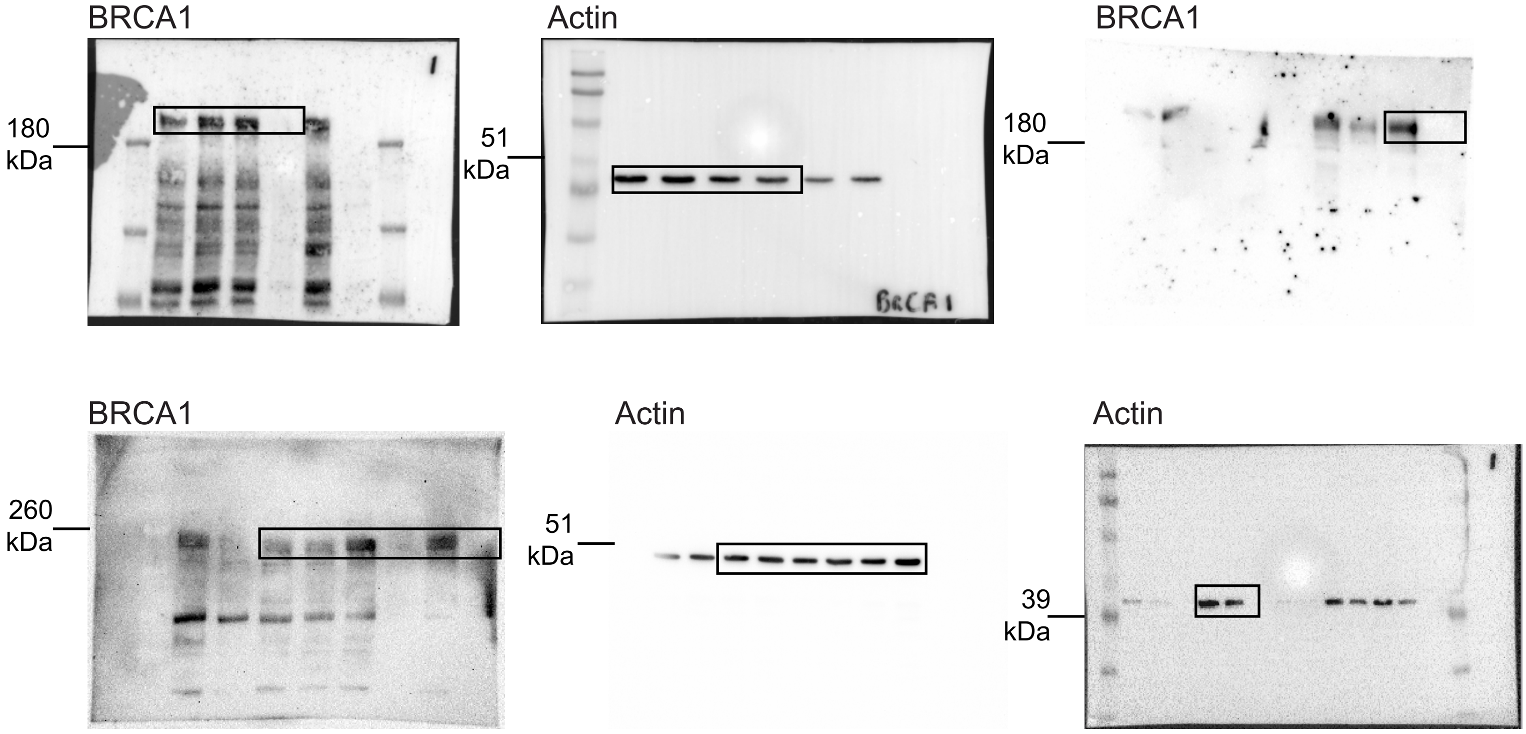

Figure S6B

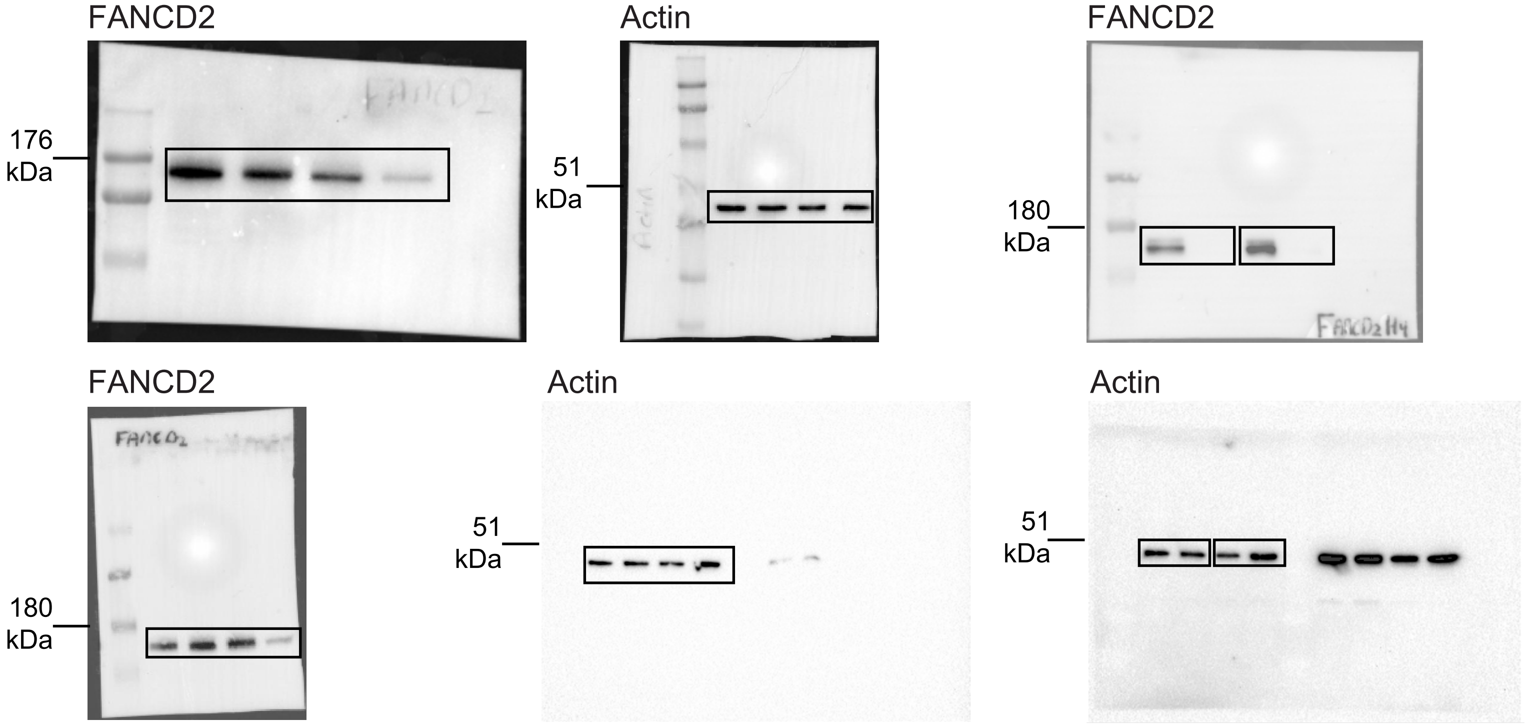

Supplementary Figure 13 - Uncropped western blots

Figure S7C

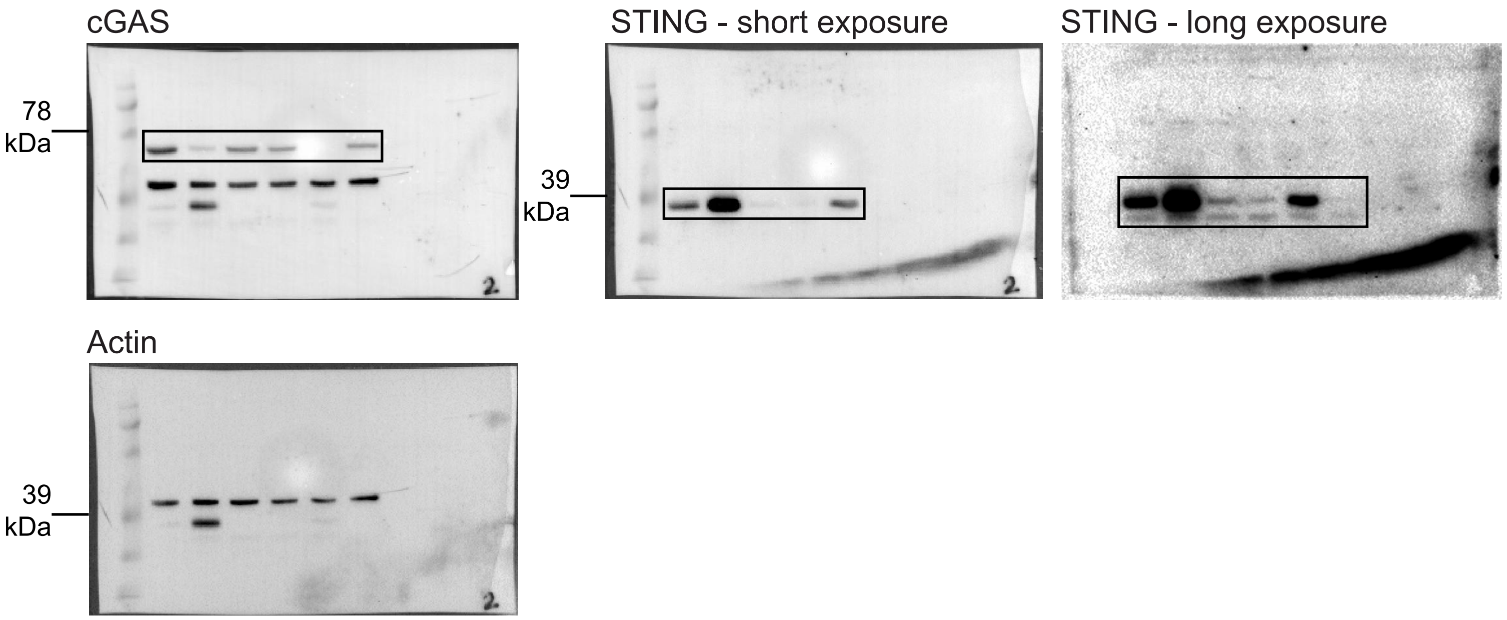

Figure S7D

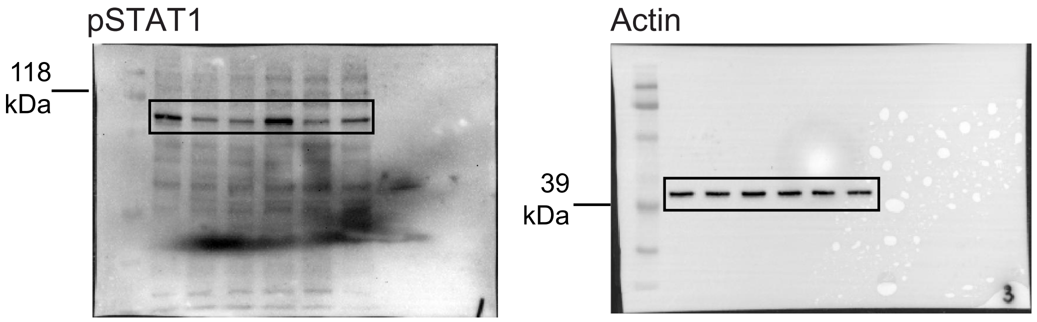

Figure S7H

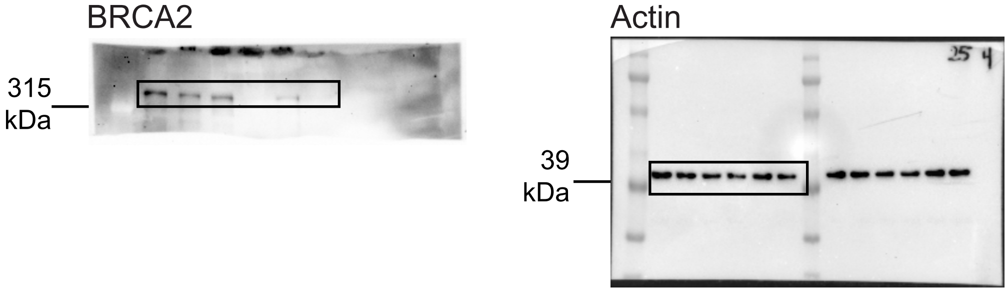

Figure S7I

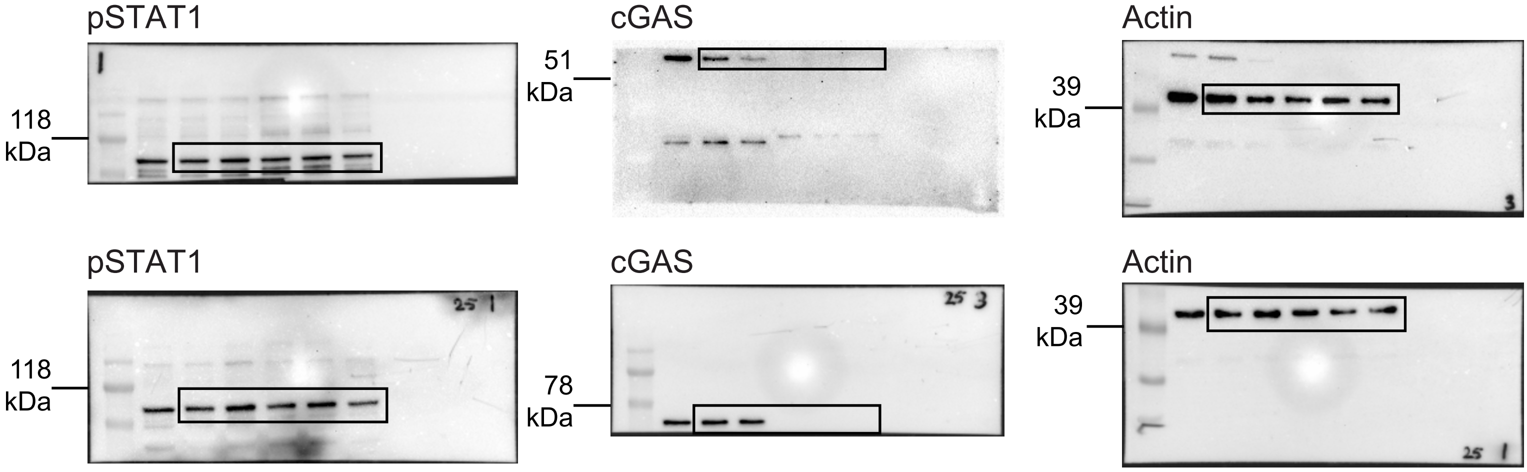

Supplement: Supplementary file 1 — Supplementary Information [file 41467_2018_7927_MOESM1_ESM.pdf]
