# Supplementary material for: BRCA2 deficiency instigates cGAS-mediated inflammatory signaling and confers sensitivity to tumor necrosis factor-alpha-mediated cytotoxicity
Source: Nat Commun. 2019 Jan 9;10:100. doi: 10.1038/s41467-018-07927-y (PMC6327059; doi:10.1038/s41467-018-07927-y)
Supplement: Supplementary file 3 — Description of Additional Supplementary Files [file 41467_2018_7927_MOESM3_ESM.pdf]

Description of Additional Supplementary Files

File Name: Supplementary Data 1
Description: Gene trap integration sites mapped to individual gene loci in KBM-7 shBRCA2#2 cells. Numbers of forward ('sense') and reverse ('anti-sense) integrations are indicated.

File Name: Supplementary Data 2
Description: Tab 1: Numbers of gene-trap insertions per gene in BRCA2-depleted KBM-7 cells versus control KBM-7 cells. Tab 2: Numbers of gene-trap insertions per gene in Eg5-depleted KBM-7 cells versus control KBM-7 cells

File Name: Supplementary Data 3
Description: Log2 ratios of identified peptides by SILAC mass spectrometry in BRCA2-depleted BT-549 and HCC38 cells are indicated.

File Name: Supplementary Data 4
Description: Statistical analysis of cell viability assays in Figures 4, 6, and Supplementary Figures 5, 6 and 7.
