# Supplementary material for: BRCA2 deficiency instigates cGAS-mediated inflammatory signaling and confers sensitivity to tumor necrosis factor-alpha-mediated cytotoxicity
Source: Nat Commun. 2019 Jan 9;10:100. doi: 10.1038/s41467-018-07927-y (PMC6327059; doi:10.1038/s41467-018-07927-y)
Supplement: Supplementary file 8 — Reporting Summary [file 41467_2018_7927_MOESM8_ESM.pdf]

nature research

Corresponding author(s): NCOMMS-17-29551A

Reporting Summary

Nature Research wishes to improve the reproducibility of the work that we publish. This form provides structure for consistency and transparency in reporting. For further information on Nature Research policies, see Authors & Referees and the Editorial Policy Checklist.

Statistical parameters

When statistical analyses are reported, confirm that the following items are present in the relevant location (e.g. figure legend, table legend, main text, or Methods section).

n/a	Confirmed	
☐	☒	The exact sample size (n) for each experimental group/condition, given as a discrete number and unit of measurement
☐	☒	An indication of whether measurements were taken from distinct samples or whether the same sample was measured repeatedly
☐	☒	The statistical test(s) used AND whether they are one- or two-sided<br>*Only common tests should be described solely by name; describe more complex techniques in the Methods section.*
☒	☐	A description of all covariates tested
☒	☐	A description of any assumptions or corrections, such as tests of normality and adjustment for multiple comparisons
☐	☒	A full description of the statistics including central tendency (e.g. means) or other basic estimates (e.g. regression coefficient) AND variation (e.g. standard deviation) or associated estimates of uncertainty (e.g. confidence intervals)
☐	☒	For null hypothesis testing, the test statistic (e.g. F, t, r) with confidence intervals, effect sizes, degrees of freedom and P value noted<br>*Give P values as exact values whenever suitable.*
☒	☐	For Bayesian analysis, information on the choice of priors and Markov chain Monte Carlo settings
☒	☐	For hierarchical and complex designs, identification of the appropriate level for tests and full reporting of outcomes
☒	☐	Estimates of effect sizes (e.g. Cohen's d, Pearson's r), indicating how they were calculated
☐	☒	Clearly defined error bars<br>*State explicitly what error bars represent (e.g. SD, SE, CI)*

Our web collection on statistics for biologists may be useful.

Software and code

Policy information about availability of computer code

Data collection	For indicated genes in Supplemental Figure S2A, mRNA expression levels from the Ovarian Serous Cystadenocarcinoma TCGA dataset were retrieved from cBioportal. Only tumors with sequencing and CNA data (316 samples) were used, and were subclassified in 'BRCA2 wildtype' (193 samples) and 'germline BRCA2 mutant' (25 samples).
Data analysis	No specific software was used

For manuscripts utilizing custom algorithms or software that are central to the research but not yet described in published literature, software must be made available to editors/reviewers upon request. We strongly encourage code deposition in a community repository (e.g. GitHub). See the Nature Research guidelines for submitting code & software for further information.

Data

Policy information about availability of data

All manuscripts must include a data availability statement. This statement should provide the following information, where applicable:

- Accession codes, unique identifiers, or web links for publicly available datasets
- A list of figures that have associated raw data
- A description of any restrictions on data availability

RNA sequencing data is accessible at the GEO repository, under accession number GSE116943. The mass spectrometry data have been deposited to the

ProteomeXchange Consortium via the PRIDE partner repository with the dataset identifier PXD007253 (BT-549) and PX284973 (HCC38). DNA sequencing data is available at the NCBI short read archive (PRJNA299537).

Field-specific reporting

Please select the best fit for your research. If you are not sure, read the appropriate sections before making your selection.

☒ Life sciences ☐ Behavioural & social sciences ☐ Ecological, evolutionary & environmental sciences

For a reference copy of the document with all sections, see nature.com/authors/policies/ReportingSummary-flat.pdf

Life sciences study design

All studies must disclose on these points even when the disclosure is negative.

Sample size: For all experiments, sample size pre-calculation was not performed. In general, experiments were performed 3 times (n=3) including technical replicates whenever possible to compensate for experimental variation. These numbers are sufficient to obtain statistical differences or effect sizes.

Data exclusions: No data were excluded from the analyses.

Replication: To compensate for experimental variation, experiments were performed at least three times including technical replicates. There are no findings that were not replicated.

Randomization: For our experiments, no randomization was applied.

Blinding: Investigators were not blinded to group allocation or data analysis.

Reporting for specific materials, systems and methods

Materials & experimental systems

n/a	Involved in the study	
☒	☐	Unique biological materials
☐	☒	Antibodies
☐	☒	Eukaryotic cell lines
☒	☐	Palaeontology
☒	☐	Animals and other organisms
☒	☐	Human research participants

Methods

n/a	Involved in the study	
☒	☐	ChIP-seq
☐	☒	Flow cytometry
☒	☐	MRI-based neuroimaging

Antibodies

Antibodies used: BRCA2 (Calbiochem, #OP95), TNFR1 (Cell Signaling, #3736; Santa Cruz, sc-8436; Abcam, #19140), SAM68 (Santa Cruz, sc-333), BRCA1 (Cell Signaling, #9010), FANCD2 (Santa Cruz, sc-20022), phospho-JNK (Cell Signaling, #9251), phospho-p38 (Cell Signaling, #4511), cleaved-PARP (Cell Signaling, #5625), γH2AX (Cell Signaling, #9718), phospho-STAT1 (Cell Signaling, #9167 & #8826), HSP90 (Santa Cruz, #sc-69703), cGAS (Cell Signaling, #15102), STING (Cell Signaling, #13647), Caspase-3 (Cell Signaling, #9662), Caspase-8 (Enzo, #ALX-804-242), Caspase-9 (Cell Signaling, #9502), beta-Actin (MP Biochemicals, #69100), RAD51 (GeneTex, GTX70230), anti-BrdU (AbD Serotec).

Validation: For all antibodies used, validation statements and relevant citations can be found on the manufacturere's website. Further validation of antibodies is supported by western blotting of knock-down experiments in the manuscript (e.g. Figure 1A, Figure 6E, Figure S2E, Figure S4A,C,F)

Eukaryotic cell lines

Policy information about cell lines

Cell line source(s): KBM-7, BT-549, HCC38, MDA-MB-231, HEK293T cells were obtained from ATCC. DLD-1 human colorectal adenocarcinoma cells were from Horizon (Cambridge, UK). Mouse embryonic fibroblasts harboring the Brca2sko allele were a kind gift from Jos Jonkers and Peter Bouwman (Netherlands Cancer Institute, Amsterdam, the Netherlands).

Authentication	Cell lines were regulary authenticated using STR profiling.
Mycoplasma contamination	All cell lines used were regularly tested negative for mycoplasma contamination.
Commonly misidentified lines (See ICLAC register)	No commonly misidentified cell lines were used.

Flow Cytometry

Plots

Confirm that:

☒ The axis labels state the marker and fluorochrome used (e.g. CD4-FITC).

☒ The axis scales are clearly visible. Include numbers along axes only for bottom left plot of group (a 'group' is an analysis of identical markers).

☒ All plots are contour plots with outliers or pseudocolor plots.

☒ A numerical value for number of cells or percentage (with statistics) is provided.

Methodology

Sample preparation	Cells used for flow cytometry (KBM-7, MDA-MB-231, BT-549, MEFs) were pre-treated with doxycyline or HU when indicated. To measure changes in mCherry positivity, cells were harvested and measured immediately by flow cytometry. Otherwise, cells were harvested washed and fixed in ice-cold 70% ethanol. Cells were permeabilized and blocked with PBS-1%BSA-0.05% Tween20 or with PBS-2%BSA-0.1%Triton for 1 hour and stained with indicated antibodies in the methods section.
Instrument	LSR-II (Becton Dickinson) and FACS Calibur (Becton Dickinson)
Software	Samples on the LSR-II were acquired using DIVA 8.0 software. Samples on the FACS Calibur were acquired using CellQuest sofyware. All samples were analyzed using FlowJo.
Cell population abundance	For all experiments, at least 10,000 cells were measured to obtain relevant numbers after gating.
Gating strategy	Living cells were initially gated on the FSC/SSC plots. Boundaries between positive and negative populations were determined with samples without antibody staining to set gates.

☒ Tick this box to confirm that a figure exemplifying the gating strategy is provided in the Supplementary Information.
